# Locally advanced breast cancer is the number of dissected nodes after neoadjuvant chemotherapy important?

**DOI:** 10.3332/ecancer.2026.2070

**Published:** 2026-01-29

**Authors:** Josepmilly Del Valle Peña Colmenares, Wladimir José Villegas Rodríguez, Osama Bahsas Zaky, Carlos Eduardo Martínez, Douglas José Angulo Herrera

**Affiliations:** 1 General Surgeon/Oncologic Surgeon, Department of Breast Pathology, Hospital Oncology Service of the Venezuelan Institute of Social Security (SOH-IVSS), Caracas, Venezuela; 2 School of Statistics and Actuarial Sciences, Central University of Venezuela (UCV), Caracas 1040, Venezuela; a https://orcid.org/0000-0002-1114-6289; b https://orcid.org/0000-0001-8999-9751; c https://orcid.org/0000-0003-2051-7077; d https://orcid.org/0009-0006-1714-0984; e https://orcid.org/0009-0003-5506-0297

**Keywords:** breast cancer, axillary lymphadenectomy, neoadjuvant chemotherapy

## Abstract

**Objective:**

To assess whether the number of lymph nodes (LN) in breast cancer (BC) patients undergoing axillary dissection (AD) after neoadjuvant chemotherapy affects disease-free survival (DFS) and overall survival (OS).

**Methods:**

Descriptive, retrospective, longitudinal cut-off study (2011–2020).

**Results:**

391 patients, 176 patients in the <10 LNs group and 215 ≥10 dissected LGs. The mean number of dissected nodes was 6.2 and 13.8 in the < or ≥ 10 LN groups, respectively. The <10 LN group had a higher proportion of stage IIIB (*p* = 0.012) and ypN0 (*p* = 0.001) patients and higher frequency in the phenotypes: luminal A 23.5%, TN 24.1% and HER 2 18.7% when compared with patients with ≥10 LN. Patients with ≥10 LN retrieved had a higher mean OS compared to the group of patients <10 LN with no statistical association (*p* = 0.184) (hazard ratio = 1.91 95% CI: 0.73–4.98) and a survival probability at 120 months (both groups) of 96.2%. There was also no statistical difference in the DFS when comparing the two groups of patients, indicating that the number of nodes removed is not associated with a differential risk of relapse, with a survival probability at 120 months of 63.3%.

**Conclusion:**

The results of the study indicate that the number of nodes removed during AD does not affect survival (OS and EFS) in patients with neoadjuvant (ypN0/ypN+) BC. Axillary staging remains a key factor in the management of BC; therefore, an individualised approach considering the response to triple negative breast cancer and tumour burden in therapeutic decision making is recommended.

## Introduction

Axillary lymph node (LN) metastasis remains an important prognostic factor in breast cancer (BC). The Danish Breast Cancer Cooperative Group [1] studied 13,851 patients with a diagnosis of BC more than 30 years ago, concluding that the number of LGs removed had to be at least 10 LGs to exclude misclassification of LN-positive patients as node negative and this in turn correlated with a very significant improvement in prognosis. Kiricuta and Tausch [2] and Veronesi *et al* [3], also evaluated the LN thresholds needed to ensure a node-negative axilla (10 LN). Insufficient dissection can lead to inaccurate staging of the axilla, and removal of more nodes allows a more accurate determination of the node LN status [2]. It was then established in the 1990s that the minimum number of nodes to be retrieved for more accurate staging of the axilla should include ten nodes.

Axillary dissection (AD) was considered diagnostic, prognostic and therapeutic in the 1980s and 1990s; however, it is not without complications that can affect the quality of life of patients. Although the prognostic value of axillary staging for MCC has been well established and AD has been considered the standard procedure in patients with MCC, with or without positive nodes, until the end of the last century. However, time and different clinical trials have demonstrated a diverse range of possibilities in different scenarios, such as: patients with positive nodes that do not warrant dissection (ACOZ0011) [4] or patients receiving neoadjuvant chemotherapy (NCT), the latter being a reason for discussion about the different staging techniques and/or treatment to the axilla in patients with negative nodes after NCT.

However, at present, the ideal number of nodes dissected in BC patients with residual disease in the axilla after neoadjuvant therapy is not defined. The National Comprehensive Cancer Network defines adequate AD as recovery of ≥10 LGs to accurately stage the axilla [5] and recommends performing AD at Berg levels I and II, in patients with residual disease in the axilla post-NCT [6]. We are currently awaiting the results of the various ongoing studies that define the treatment of the axilla in the presence of residual disease limited to the sentinel node in patients receiving triple negative breast cancer (TNBC).

The aim of this study was to evaluate whether the number of LGs removed in patients with BC who received TNBC with subsequent AD affects disease-free survival (DFS) and overall survival (OS).

## Methods

Descriptive, retrospective, longitudinal study of 2,633 patients diagnosed with BC between January 2011 and December 2020, treated in the Breast Pathology and Medical Oncology Services of the Hospital Oncology Service of the Venezuelan Institute of Social Security (SOH-IVSS), 1,517 were operated on and 391 met the inclusion criteria: 1. Female patients over 18 years of age with histological and immunohistochemical diagnosis of stage II and III BC, with cytology demonstrated as positive axillary node and/or axillary ultrasound with suspicion of malignancy. The tumours were classified according to the parameters of the American Joint Committee on Cancer (AJCC eighth edition) [7]. 2. They had received standard NCT treatment, approved by the Medical Oncology Service and surgery in the Breast Pathology Service of the SOH-IVSS. 3. The patients underwent DA dissection after NCT. Evaluation of the surgical specimen by anatomic pathology specialists, using the Miller and Payne classification [8, 9] in all specimens. Patients' complete pathological response (PCR) was defined as: ypT0ypN0 or ypTisypN0 and by Miller and Payne classification (Grade V for complete breast response/ A and D for axillary response). 5. Two groups were analysed for the study, one group had <10 LN after DA and the other had ≥10 LN. Of note, the sample reviewed included patients with locally advanced BC and where selective sentinel node lymphadenectomy post-NCT was in the study protocol in our centre from 2014 to 2019 (all patients had DA completed). In total, 84.65% of the patients received adjuvant radiotherapy (RT).

In the present study, the Helsinki Declaration belonging to the World Medical Association was considered as the ethical basis. This research protocol was approved by the Ethics Committee of the IVSS SOH [10].

### Objective

To evaluate whether the number of LN in patients with MS undergoing DA after NCT affects EFS and OS.

### Specific objectives

To determine whether the number of nodes retrieved (<10 or ≥10 LN) is related to recurrence when performing AD. 2. To identify the clinical and pathological characteristics of the patients in the study group. 3. To identify whether in patients with (ypN+) or without (ypN0) nodal residual disease, the number of retrieved LGs affects OS and DFS.

### Statistical analysis

The mean and standard deviation of the quantitative variables were calculated; in the case of qualitative variables, their frequencies and percentages were calculated. The results were arranged in unidimensional tables and contingency tables. Differences between groups, <10 nodes and ≥nodes, in the case of qualitative variables, were performed with Pearson's chi-square test; in the case of quantitative variables, the Student's *t*-test for independent samples was applied, after normality check, with the Shapiro–Wilk test. OS and DFS were determined using the Kaplan–Meier procedure, and the differences between the survival curves were determined using the log-rank test. A value was considered statistically significant if *p* < 0.05. Data were tabulated with R Studio version 2024.12.0+467. The hazard ratio (HR) was calculated using a Cox proportional hazards model. Relevant independent variables were adjusted for and model assumptions, including proportional hazards, were checked. The HR was interpreted as the change in the risk of event occurrence between groups, holding all other variables constant. A value was considered statistically significant if *p* < 0.05. Data were tabulated with R Studio version 2024.12.0+467.

## Results

Thirty-nine hundred and ninety-one patients met the inclusion criteria. The mean age at disease diagnosis was 51 years ± 11 (ranging from 25 to 87 years), 176 patients in the <10 LN group and 215 ≥ 10 dissected nodes. The average number of dissected LGs was 6.32 and 13.8 in the groups and the proportion of positive LGs 0.23 and 0.20< or ≥ 10 LGs, respectively. The rate of PCR 19.3%, PCR in the breast (CPRR) 22.7% and axillary pathological response (APR) 55.7% when <10 LN were retrieved, when dissected or retrieved ≥10 LN the found rates of PCR, CPRR and APR reported 16.3%, 19.5% and 45.1%, respectively.

Table 1 shows the characteristics of the patients according to the number of LGs. The three patients with stage IIIC in the <10 LN group corresponded to the AJCC classification before NCT.

In the analysis of mortality and recurrence as a function of the number of nodes removed, it was identified that the mortality rate was higher in patients with ≥10 resected nodes, with 12 (5.6%) deaths compared to 5 (2.8%) in the group with <10 nodes, although the difference was not statistically significant (*p* = 0.186). The proportion of relapses (local, regional and/or distant) was similar in the two groups; in the <10 nodes group, it was 17.6% with 13.6% distant relapses, while in the ≥10 group 16.7% relapses, of which 13.5% included distant relapses with no statistical association (*p* = 0.820)

**Figure 1.**
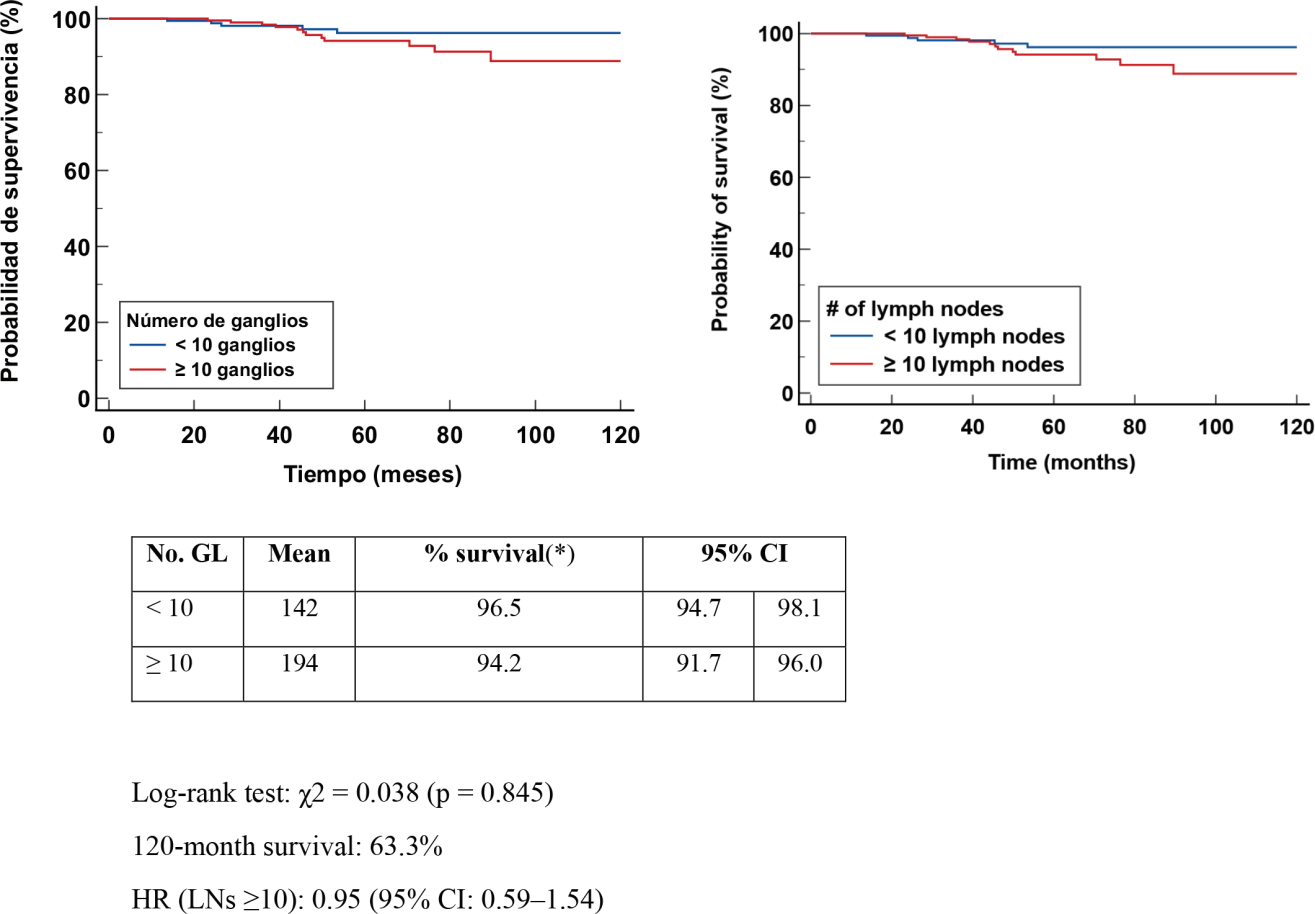
Kaplan Meier OS curve according to LN number (No.).

Kaplan-Meier OS curve analysis evaluated the number of salvaged LGs. The log-rank test yielded a c^2^ = 1.765 (*p* = 0.184). Clinical interpretation of the results indicates that there was no statistical association in OS in patients with BC who received NCT to whom DA < or ≥ 10 LN was performed. Patients with ≥10 LN had a longer median survival compared to the group of patients <10 LN, with no statistical association and both groups with a 120-month survival probability of 96.2%. The HR for patients with ≥10 nodes removed was 1.91 (95% CI: 0.73–4.98) Figure 1.

**Figure 2.**
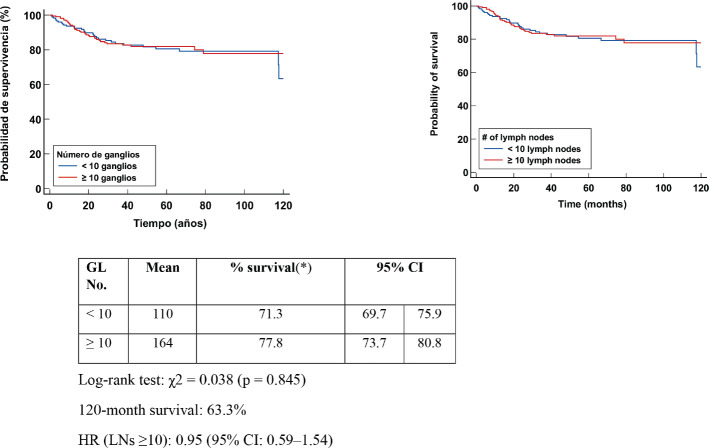
Kaplan Meier DFS curve according to LN number.

The Kaplan–Meier DFS curve analysis evaluated the number of LGs. The log-rank test yielded a c^2^ = 0.038 (*p* = 0.845). Clinical interpretation of the results indicated that DFS in patients with ≥10 had a mean 164 months at the end of follow-up; however, there was no statistical difference when compared with DA <10 LN. The HR in the group with ≥10 nodes was 0.95 (95% CI: 0.59–1.54), indicating that the number of nodes removed was not associated with a differential risk of relapse. This reinforces the need for a comprehensive assessment of axillary status and tumour biomarkers to better predict disease progression Figure 2.

### Patients with post-NCT residual nodal disease (ypN1,ypN2,ypN3)

**Figure 3.**
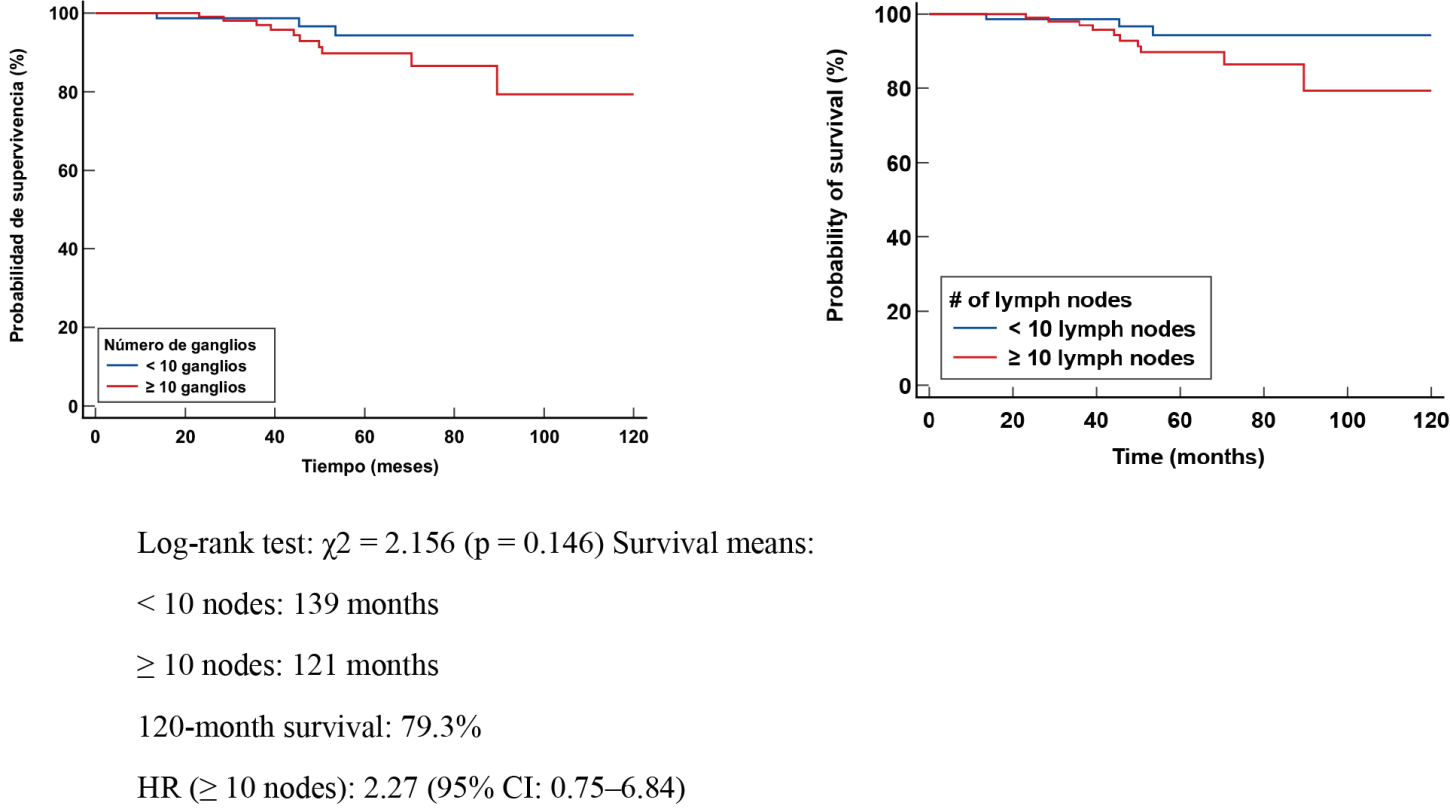
Kaplan Meier OS curve according to number of positive LN (+).

In patients with lymphonodal metastases or residual nodal disease, the median OS was 139 months in those with <10 nodes removed and 121 months in those with ≥10 nodes. Survival at 120 months was 79.3%. No significant differences were identified in the log-rank test (χ² = 2.156, *p* = 0.146). However, the HR in the group with ≥10 nodes was 2.27 (95% CI: 0.75–6.84), suggesting a possible higher mortality risk in this group. This could be explained by the larger tumour volume present in these cases or by the persistence of residual disease after TNBC (Figure 3).

**Figure 4.**
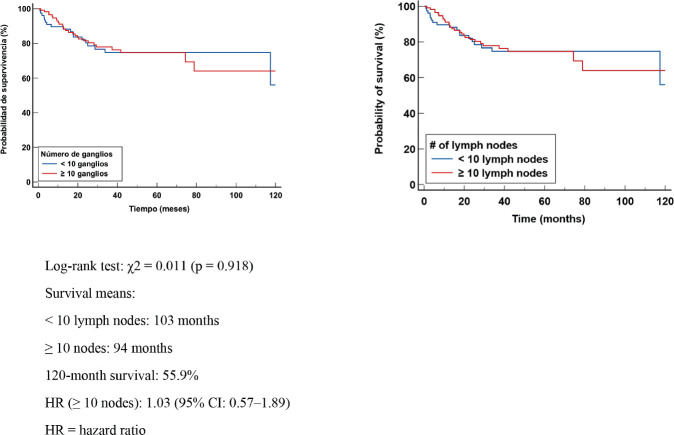
Kaplan Meier DFS curve according to number of positive (+) GLs.

In patients with lymphnodal involvement (ypN+), median EFS was 103 months in those with <10 nodes removed and 94 months in those with ≥10 nodes. Survival at 120 months was 55.9%. The log-rank test showed no significant difference (χ² = 0.011, *p* = 0.918). The HR was 1.03 (95% CI: 0.57–1.89) (Figure 4). The absence of differences in DFS suggests that the number of excised LGs does not significantly modify the risk of relapse in patients with axillary involvement. These findings reinforce the importance of tumour biological factors and response to NCT as major determinants of prognosis.

### Patients with no residual nodal disease following NCT

**Figure 5.**
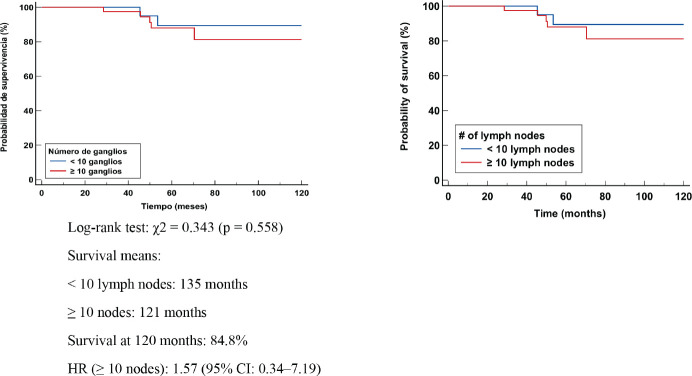
Kaplan Meier OS curve according to number of negative LN (-).

**Figure 6.**
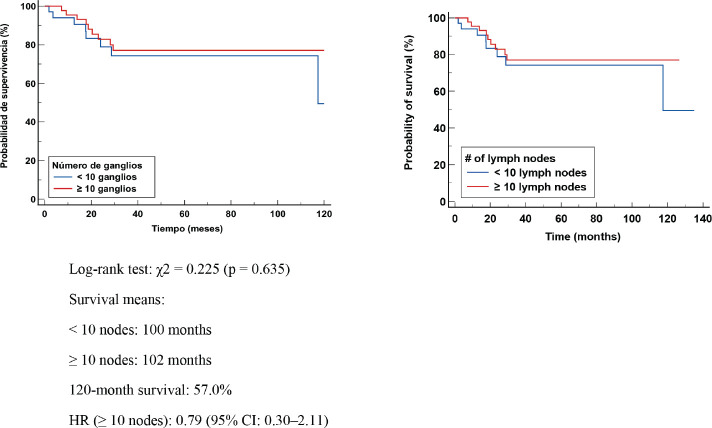
Kaplan Meier DFS curve according to negative LN numbers (-).

In patients with axillary pathologic complete response (APCR), no significant difference in OS or EFS was observed. These results suggest that in patients with ypN0 or APCR, the number of retrieved LGs does not appear to significantly influence OS, supporting the possibility of performing axillary de-escalation or reduced axillary surgery in this group of patients Figure 5. The presence of a PRC suggests that the number of LNs removed does not significantly influence the risk of relapse in patients without axillary involvement Figure 6.

## Discussion

The number of positive GLs is strongly associated with the AJCC *N* (nodal) stage, having positive GLs is a critical factor in determining strategies in adjuvant systemic therapy [11, 12]. DA still continues to be the treatment of choice for patients with post-CTN nodal residual disease.

Rosenberger *et al* [13], in their cohort study including 129,685 patients from the National Cancer Data Base divided the sample into two groups according to treatment sequence: initial surgery 102,200 (78.8%) or NCT 27,485 (21.2%) to identify the optimal yield of LN DA among LN positive patients concluding that removal of approximately 20 LGs may improve survival through more accurate nodal staging and increased use of adjuvant therapy. Wang *et al* [14], also studied the optimal threshold of LGs examined for BC patients with >3 positive LGs after modified radical mastectomy (MRM), with the threshold being number 12 for improved cancer-specific survival, in contrast to Neuman *et al* [15], who report that a recovery of less than ten nodes in patients who have received TNBC may not necessarily imply inadequate surgical staging.

In our review, we evaluated oncologic outcomes in relation to the number of resected nodes (<10 or ≥ 10) after AD in patients with ALMC who received TNBC, in the Kaplan Meier analysis there was no significant difference in relation to the number of resected nodes in terms of EFS and OS; as Choi *et al* [16], who in their research, did not find statistical significance in OS between the two groups studied (<10 and ≥10 dissected nodes). In our study, patients with residual nodal disease, the removal of ≥ 10 nodes seems to be associated with a tendency to worse prognosis, although without statistical significance. This suggests that axillary tumour volume (residual disease) and tumour biology could be more determined than the number of dissected nodes in the evolution of the disease.

However, in the subgroup reported by Choi *et al* [13, 16], patients ypN0 and ypN2, the group with <10 nodes had a lower SCE than the group with ≥10 nodes and Rosenberger *et al* [13], retrieving less than 8 LN subsequent NCT decreased OS, which increased (OS) with retrieval between 8 and 22 LN, high versus low LN retrieval was also associated with a slightly higher receipt of adjuvant RT (*p* = 0.002).

In our study there was no statistical association between the number of retrieved LGs and OS in patients who were LN negative (ypN0) which could be interpreted that APCR patients the number of retrieved LGs does not modify OS, which supports reduced axillary surgery in this type of patients in contrast to the data provided by Chen *et al* [17], whose findings associated better OS with greater retrieval **≥**10 LGs, even in a population of women with pathologically negative nodes who received TNBC followed by MRM. However, some authors describe that the number of dissected LGs in BC patients who receive TNBC may be lower than those who do not and this may not always be attributed to inadequate AD and it should be considered that TNBC may reduce the number of LGs [15, 18, 19].

A higher number of LGs examined after MRM could provide more accurate information on TNM stage and adjuvant therapy. However, a higher number of LGs examined is associated with a higher risk of adverse outcomes [20, 21]. Several authors [13, 14, 17] report that obtaining a greater number of nodes on DA in patients with stages I, II or III post-TNM stage I, II or III BC with negative or positive LNs increases EFS and/or OS and that this is probably due to a reduction in false-negative staging, as well as a greater extent of DA may be an indicator of more aggressive overall care with increased receipt of adjuvant RT and/or chemotherapy, but as well defined by Roseberger *et al* [13], does not interpret these data to suggest a therapeutic survival benefit due to additional axillary clearance, but in the possible influence on adjuvant treatment decision making. In our review, no significant difference was found; all our patients were advanced stages and most of them received adjuvant treatment (systemic therapy and/or RT), where the number of dissected LGs (<10 or ≥10) did not influence OS, EFS or the frequency of local, regional and/or distant recurrence rate.

When dissecting < However, the exact percentage of underestimation when less than ten nodes are obtained is not expressly quantified [1, 2, 22, 23] in the aforementioned works, in general the importance of recovering as many nodes as possible to reduce the rate of false negatives in axillary staging is described, it would be thoughtless or daring to conclude that regional or distant recurrence in these patients is inversely proportional only to the number of resected LN, since MS is considered a systemic disease and it is the intratumoural heterogeneity, the biomolecular differences in the tumour microenvironment, the use of targeted therapies and the various randomised trials that have allowed us to acquire a better understanding of the various characteristics of tumour behaviour.

In the same vein, when reviewing relapse rates in various series with less than ten retrieved nodes, Neuman *et al* [15] stated that axillary recurrence is not determined in patients during their 72-month follow-up after removal of <10 LN and proposed that the surgical technique they applied was sufficient. Uyan *et al* [18] did not observe axillary recurrence in any patient who received TNBC and had <10 LN removed during their 36-month follow-up. In our review, the loco-regional relapse rate (<10 retrieved LN) was 4.5% at the end of 120 months follow-up, as already mentioned, with no statistical association when compared to the group of >10 retrieved LN and no influence on survival.

Having more positive nodes implies a higher probability of local and distant relapse, higher stage disease, resecting more nodes or ‘increasing’ the number of nodes retrieved in the AD in LN positive patients will probably reduce the tumour burden and achieve regional control, but it will not increase survival. There are currently a large number of prospective trials involving the use of new chemotherapeutic agents, monoclonal antibodies and/or biological agents in smaller and smaller tumours in search of a better pathologic response rate. A la lustre of new studies, most or nearly all tumours will receive some form of targeted therapy for a specific tumour subtype, which proportionally increases the number of patients receiving neoadjuvant systemic therapy, more selective sentinel node lymphadenectomies, more targeted ADs and less DA.

If there are positive LNs post-NCT without evidence of distant progression, our recommendation, like most authors so far, remains DA of levels I and II.

However, it is important to note that the presence of extensive axillary positive LGs (N2 or N3a) is related to aggressive tumour biology and therefore may indicate the need for more systemic treatment and/or RT and not necessarily more extensive surgery, as this does not compensate for adverse tumour biology, making it clear that in patients with positive LGs after TNCT, DA is still part of the multidisciplinary treatment.

## Conclusion

The results of the study indicate that the number of nodes removed during DA is not significantly associated with OS or EFS in TNBC patients treated with TNBC. This suggests that axillary tumour burden and tumour biology may be more determined than the number of nodes dissected during disease progression.

The goal of BC surgery is currently to reduce treatment without affecting survival. Time and trials demonstrated that RT replaces or is as effective as AD in patients with limited sentinel LN involvement; however, there is still no evidence on the results of de-escalated axillary staging after NCT (LN positive). The ingrained ideology that more surgery is always better over time is probably changing, PCR increasingly translates into a prognostic factor and reduced axillary surgery becomes more relevant (so the number of resected nodes in ycN0 patients would not be the main target). Ongoing trials such as: ALLIANCE 202, ADARNAT trial [24] will confirm or not the efficacy and safety of axillary RT on DA in patients with limited GC involvement after NCT, probably give us the answer of axillary de-escalation or escalation in patients who receive NCT; however, until we have more evidence, patients with positive LN following neoadjuvant, DA remains a key factor in the management of BC, an individualised approach is recommended, considering the response to TNBC and tumour burden in therapeutic decision making.

## Limitations

1. It should be noted that our hospital is a specialised oncology centre and our Breast Pathology service is made up of specialists in oncologic surgery and breast pathology, it is a teaching hospital setting, training oncologic surgery residents and although there are reports that there are higher rates of recovery of more than 10 LN from surgeons with an academic affiliation or in a teaching hospital setting, we have not performed such a review to compare whether the AD performed is higher or lower when performed by residents in training and monitored by attendings in our service and if there is any significant difference, so we do not know if this could have influenced the results. 2. This study was a retrospective review, from a single oncology centre, with a small sample, so the distribution of patients could be uneven, present certain population differences among the patients analysed and have some effect on the results of regional control. 3. We are aware that at the moment of performing the DA several factors influence, including the patient, the surgery, the processing of the anatomic pathology specimen, the NCT as some authors describe it, we do not have a central review for histopathological studies, so we do not rule out that these factors have influenced our review, besides being a retrospective study. 4. The data are not generalisable because the sample was limited to a single hospital centre, despite the fact that our hospital is considered a national reference for the treatment of MS.

## Conflicts of interest

The authors declare that there is no conflicts of interest that could have affected the results of the study.

## Funding

No financial support was received for the writing of this article, data collection or analysis or in the preparation or approval of the current manuscript.

## Disclosure

The authors have no financial interest to declare in relation to the disclosure of the contents of this article, as their only interest is focused on opening a line of research in our country on the subject matter of the study.

## Figures and Tables

**Table 1.** Distribution of patients by number of LNs and clinical and surgical variables.

	< 10 LNs		≥ 10 LNs		
Variables	No.	%	No.	%	*p*
*n*	176		215		-
Age (years) (*)	51 ± 10		52 ± 11		0.478
BMI	27.9 ± 4.7		27.4 ± 4.7		0.263
Stages					**0.012**
IIA	2	1.1	4	1.9	
IIB	12	6.8	16	7.4	
IIIA	65	36.9	77	35.8	
IIIB	94	53.4	96	44.7	
IIIC	3	1.7	22	10.2	
Histological type					0.528
Ductal	156	88.6	186	86.5	
Lobular	15	8.5	18	8.4	
Others	5	2.8	11	5.1	
Menopause					0.663
Yes	96	54.5	122	56.7	
No	80	45.5	93	42.8	
Histological grade					0.806
G1	19	10.8	19	8.8	
G2	112	63.6	139	64.7	
G3	45	25.6	57	26.5	
RE					**0.059**
Positive	99	56.3	141	65.6	
Negative	77	43.8	74	34.4	
RP					0.964
Positive	88	50.0	108	50.2	
Negative	88	50.0	107	49.8	
HER-2					0.164
Positive	58	33.0	57	26.5	
Negative	118	67.0	158	73.5	
Ki67					0.899
Low	66	37.5	82	38.2	
High	100	56.9	123	57.2	
NR	10	5.6	10	4.6	
Tumour phenotype					**0.050**
Luminal A	39	23.5	37	18.0	
Luminal B	41	24.7	74	36.1	
Luminal B/Her-2	25	15.1	35	17.1	
Her-2	31	18.7	22	10.7	
Triple negative	40	24.1	47	22.9	
RPM/Miller and Payne					0.6
I	34	19.3	44	20.5	
II	43	24.4	48	22.3	
III	39	22.2	45	20.9	
IV	20	11.4	36	16.8	
V	40	22.7	42	19.5	
RPA					**0.001**
yPN0	98	55.7	97	45.1	yPN0
yPN1	44	25	55	25.6	yPN1
yPN2	34	19.3	46	21.4	yPN2
yPN3	0	0	17	7.9	yPN3

RPM: pathological response in breast. RPA: pathological response in axilla

Statistical significance was < 0.05 for all the bold, single asterisk and highlighted values.
